# Molecular cloning and expression of a new gene, GON-SJTU1 in the rat testis

**DOI:** 10.1186/1477-7827-8-43

**Published:** 2010-05-12

**Authors:** Zhao-juan Yang, Ning Sun, Shu-qin Wang, Geng G Tian, Ji Wu

**Affiliations:** 1 School of Life Sciences and Biotechnology, Shanghai Jiao Tong University, Shanghai, 200240, China; 2 School of Life Science, Nanjin Normal University, Nanjin, Jiangsu 210097, China

## Abstract

**Background:**

Spermatogenesis is a complex process involving cell development, differentiation and apoptosis. This process is governed by a series of genes whose expressions are highly regulated. Male infertility can be attributed to multiple genetic defects or alterations that are related to spermatogenesis. The discovery, cloning and further functional study of genes related to spermatogenesis is of great importance to the elucidation of the molecular mechanism of spermatogenesis. It is also physiologically and pathologically significant to the therapy of male infertility.

**Methods:**

GON-SJTU1 was identified and cloned from rat testis by cDNA library screening and 3'-and 5'-RACE. The products of GON-SJTU1 were assessed by Northern and Western blotting. The expression of GON-SJTU1 was also examined by In situ hybridization and immunohistochemistry.

**Results:**

Here we identified and cloned a new gene, GON-SJTU1, with the biological process of spermatogenesis. GON-SJTU1 is highly expressed in the testis from day 1 to 15 and then decreased, suggesting that GON-SJTU1 might be a time-related gene and involved in the early stage of spermatogenesis. And the expression of GON-SJTU1 in the testis occurred in some male germ cells, particularly in gonocytes and spermatogonial stem cells.

**Conclusion:**

GON-SJTU1 may play a role in the biological process of spermatogenesis.

## Background

With the increase of infertility rates in couples, the cellular and molecular mechanisms involved in spermatogenesis have held the interest of many biologists and researchers. This complex process involves cell development, differentiation and apoptosis. Spermatogenesis is initiated by the conversion of gonocytes to spermatogonial stem cells [1-4]. The spermatogonia form the resident stem cell. It constantly divides by mitosis and differentiates through meiosis and spermiogenesis to form spermatocytes, spermatids and spermatozoa [5].

The coordinated maturation of spermatogonia, spermatocytes, and spermatids need a precise and concordant regulatory system, which comprises a large group of regulators, signals, and other factors. We recently demonstrated the importance of short-type PB-cadherin (STPB-C) in gonocyte survival, proliferation and self-renewal of spermatogonial stem cells (SSCs) [6-8]. Moreover, we found that STPB-C promoted self-renewal of SSCs via activating Janus kinase/signal transducer and activator of transcription (JAK-STAT) and phosphoinositide-3 kinase (PI3-K)/Akt, and blocking transforming growth factor (TGF)-β1 signaling [8]. Glial cell line-derived neurotrophic factor (GDNF), produced by Sertoli cells, has been reported essential for spermatogonial stem cell maintenance [9].

Stem cell factor (SCF) and its receptor c-kit consisting of a key signaling system, which appears to regulate the proliferation of spermatogonia [10]. Prabhu *et al*. demonstrated that the expression of c-kit was indeed concordant with spermatogonial differentiation [11]. Bcl-2 family members are key regulators of apoptosis. Some can promote cell survival while others support cell death [12,13]. Currently, Yan *et al*. have reported the involvement of Bcl-2 family proteins in germ cell apoptosis during rat testicular development [14]. The regulation of the SCF/c-kit system in the proliferation and survival of spermatogonia appears to be through controlling the expression of some Bcl-2 family of proteins [10,14,15].

At present, some research groups have demonstrated stage-specific expression patterns of many genes in rodents by microarray analysis, indicating that stage-regulated expression of genes is a widespread and fundamental event during spermatogenesis [16,17]. Johnson *et al*. reported that cadherins, with the exception of N-cadherin and cadherin-6, were expressed most abundantly around postnatal day 7 [18]. In addition, for all members of the PCDHα family of cadherins, expression levels were generally high at day 7 and then remained at a steady state from postnatal day 21 through adulthood [18]. This pattern is in contrast to that of STPB-C, which is expressed at high levels between days 1 and 5, with a subsequent large drop by day 10 [6], suggesting a distinct role of STPB-C in spermatogenesis. These reports have contributed valuable information for further studies into the identification of spermatogenesis-related genes.

In the present study, we identified and cloned a new gene, *GON-SJTU1*. Its mRNA contains an open reading frame of *771 *bp and is predicted to encode a protein of *256 *amino acids. We studied its expression in germ cells of rat testis at different days after birth, and speculate that GON-SJTU1 play a role in the early steps of spermatogenesis.

## Methods

### Animals

About 180 pups from different litters were obtained by mating Sprague-Dawley rats (Charles River Breeding Labs, Kingston, RI, USA). All protocols involving animals were approved by the Institutional Animal Care and Use Committee of Shanghai, in accordance with the National Research Council Guide for Care and Use of Laboratory Animals.

### Preparation of probes

Drosophila unpaired genes were previously assigned key functions in the regulation of the JAK signaling pathway and self-renewal of spermatogonial stem cells [19-21]. To clone the homolog of the unpaired gene in rats, we focused on the conserved protein sequence of Drosophila unpaired genes, and then translated the protein sequence back to a nucleotide sequence that was possibly encoded in rat. To produce screening probes, the PCR primers (forward primer: 5'-CAGGGGAATCCGACTGTTTA-3'; reverse primer: 5'-TCCGTAGGTAGGGACAGTGG-3') were designed with this purpose.

Total RNA was obtained from rat testis with TriZol reagent (Invitrogen, Carlsbad, CA, USA) as recommended. Using oligo(dT)_12-18 _primer (Life Technologies, Gaithersburg, MD), cDNA was synthesized by reverse transcription of 2 μg total RNA with Superscript II RNase H^- ^reverse transcriptase according to the manufacturer's instructions (Life Technologies). One-tenth of the reverse transcription reaction was used for PCR amplification on a thermal cycle (PTC-100 Peltier Thermal Cycler, Bio-Rad, Hercules, CA, USA) with primers (see above). The amplified DNA probe was separated by gel electrophoresis and purified using a Gel Extraction Mini Kit (Qiagen, Valencia, CA, USA).

### cDNA library screening

For the screening process, 50 ng of DNA probe obtained above was randomly labeled with [γ-32P]ATP using Prime a Gene^® ^labeling system (Promega, Madison, WI, USA). The hybridization probe was used to screen approximately 106 plaques of a rat testis cDNA library. The Lambda ZAP^® ^II Library was purchased from Stratagene. The plating of the library and subsequent plaque lift were conducted using standard procedures (Stratagene, La Jolla, CA). The plaques exhibiting positive signals were excised as pBluescript SK(-) phagemids. Ten positive clones were isolated and sequenced using the T3 and T7 promoter primers.

To clone the full-length cDNA sequence of the new gene, 3'-and 5'- RACE reaction (5' primer: 5'-GGGTCTTCCGTACGCCACAT-3'; 3' primer: 5'-GTGCTCGTGATTAATCACAGG-3') were performed using a SMART RACE cDNA amplification kit (Clontech, Palo Alto, CA, USA) with total RNA extracted from rat testis, according to the manufacturer's instructions.

### Construct

A 782 bp fragment containing the ORF of GON-SJTU1 (771bp) was generated by PCR with the primers (forward primer: 5'-TTGTCGACAATGCAGCCCAAAGCG-3'; reverse primer: 5'-CGCTCGAGTTATGTATATGAGTACAC-3') and digestion with restriction enzymes (SalI, XhoI). The fragment was then subcloned into SalI-XhoI sites of the pCMV-Myc Mammalian Expression Vector (Clontech), creating an in-frame fusion to the Myc tag (pCMV-Myc-GON-SJTU1) for transfection [22,23].

### Northern blot analysis

Total RNA isolated from various rat tissues (brain, heart, liver, lung, kidney, adrenal, ovary, and testis) and testes on days 1, 3, 5, 10, 15, 20 and 60 was obtained as described above. Northern analysis was performed as described in Wu et al. [6]. In brief, samples (10 μg RNA/lane) were run on a 1% agarose-formaldehyde gel. The samples were then transferred by capillary action to a nylon membrane (Amersham Pharmacia Biotech, Arlington Heights, IL, USA) in 20×SSC overnight. After prehybridizing for 30 min in Rapid-Hyb (Amersham Pharmacia Biotech), blots were hybridized with a ^32^P-labeled cDNA probe (a 782 bp fragment containing the ORF of GON-SJTU1, see above) produced with the Rediprime kit (Amersham Pharmacia Biotech) following the manufacturer's instructions. Blots were then washed in decreasing concentrations of SSC, 0.1% SDS. Autoradiography was performed with Kodak BioMax film (Eastman Kodak, Rochester, NY, USA). Using the StripEz DNA kit (Ambion, Austin, TX, USA), the blots were stripped and then re-probed for G3PDH with a probe produced from a commercially available rat template (Ambion).

### *In situ* hybridization

Using T3 and T7 RNA polymerase for sense and anti-sense probes respectively, DIG-labeled cRNAs of GON-SJTU1 were produced with DIG RNA labeling kit (Roche Molecular Biochemicals, Mannheim, Germany) from linearized pBluescript SK(-) vector with a GON-SJTU1 insert. Paraffin-embedded tissues of the testis on days 1, 5 and 60, were prepared. The analysis of *in situ *hybridization was performed as mentioned previously [6].

### Western immunoblotting

Lysates of rat testes on days 5, 10, 15, 20 and 60 were obtained as described previously [22]. Immunoblotting was performed using an affinity-purified polyclonal antibody raised in rabbits against the specific peptide sequence (LVEEKMKNHEGNGTD) to GON-SJTU1 by a commercial source (Invitrogen, Carlsbad, CA, USA). The identification of specificity of this polyclonal antibody was applied according the method described by Yang and Wu [8].

### Immunohistochemistry

Testis of rat on day 7 was prepared and embedded in paraffin according the method described by Yang and Wu [8]. The testis sections were dewaxed and immunofluorescence-stained specific to GON-SJTU1. The anti-GON-SJTU1 polyclonal antibody used was same as that used in the Western immunoblotting experiments. The experimental details of the immunohistochemical process were described elsewhere [22,24].

### Data analysis

The nucleotide sequence was deduced and analyzed at the NCBI website [25]. The deduced nucleotide sequence reported in this paper has been submitted to the GenBank with accession number EU605813. The protein sequence was also analyzed by NetPhos and NetPhosK Server [26,27].

For each Northern analysis, densitometry was used to obtain a numerical value for the *GON-SJTU1 *signal and *G3PDH *signal, and the final data for each sample were expressed as a ratio of these values. Each analysis was performed in triplicate, and the mean values obtained from these three experiments for each treatment group were then analyzed statistically with one-way ANOVA (analysis of variance) and a student-Newman-Keuls test.

## Results

### Cloning of rat GON-SJTU1 gene

**Figure 1:**
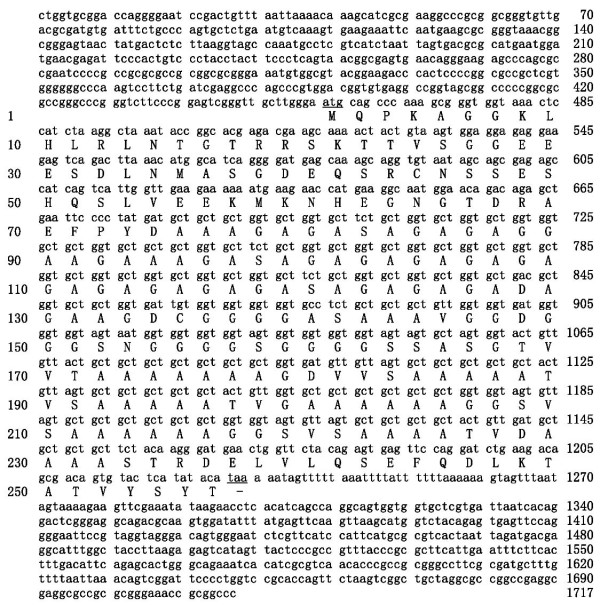
**Nucleotide sequence and predicted protein sequence of rat *GON-SJTU1***. The cDNA of *GON-SJTU1 *sequenced was approximately 1717 bp with a 771 bp ORF. The initiating codon (ATG) and the stop codon (TAA) are underlined. The predicted protein product of *GON-SJTU1 *was a polypeptide of 256 amino acids.

**Figure 2:**
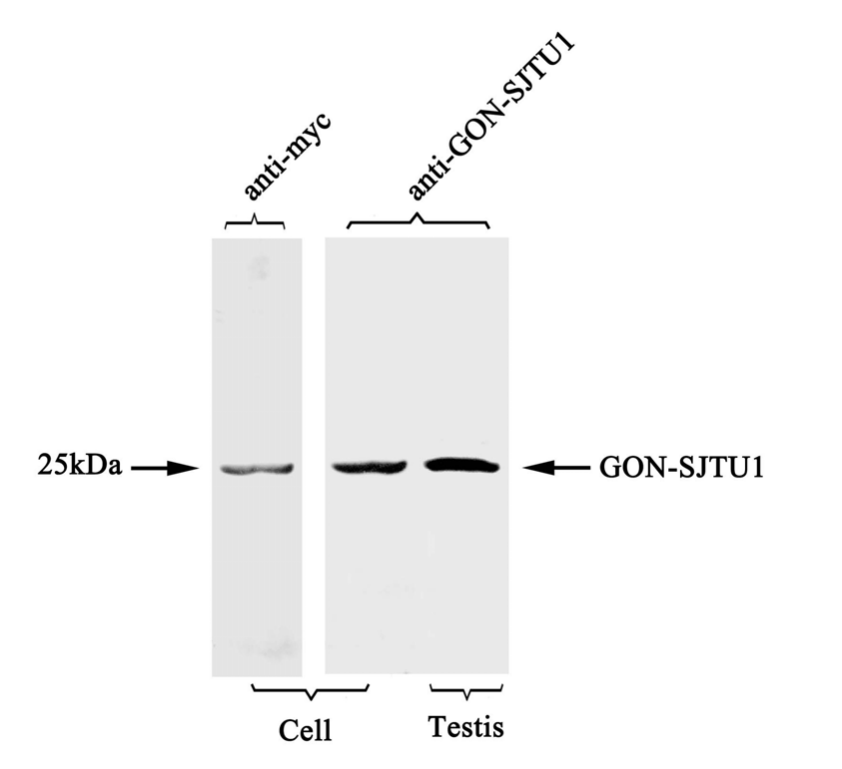
**Identification of GON-SJTU1 protein by Western blot analysis**. On immunoblots, a single prominent band of about 25 kDa in weight was recognized by anti-GON-SJTU1 antibody both in transfected cells and testis. The result of blotting analysis using anti-myc antibody corresponded with that obtained using anti-GON-SJTU1 antibody.

Using the probes (see Methods), library screening of the rat testis cDNA library resulted in identification of positive clones. The coding sequence was completed by 3'-and 5'-RACE reaction. The sequence analysis revealed a 1717 bp nucleotide sequence with an open reading frame of 771 bp predicting a 256-amino-acid protein (Figure 1). Protein of approximately 25 kDa *was *detected by Western blot analysis. The results of Western blot analysis using anti-*GON-SJTU1* antibody and that with an anti-myc antibody were consistent with data obtained using the protein from 293T cells transfected by pCMV-Myc-*GON-SJTU1* vector, indicating that the anti-*GON-SJTU1* antibody specifically recognized the *GON-SJTU1* protein (Figure 2A). In rat testis, the *GON-SJTU1* protein was also shown to be approximately 25 kDa (Figure 2B).

**Figure 3:**
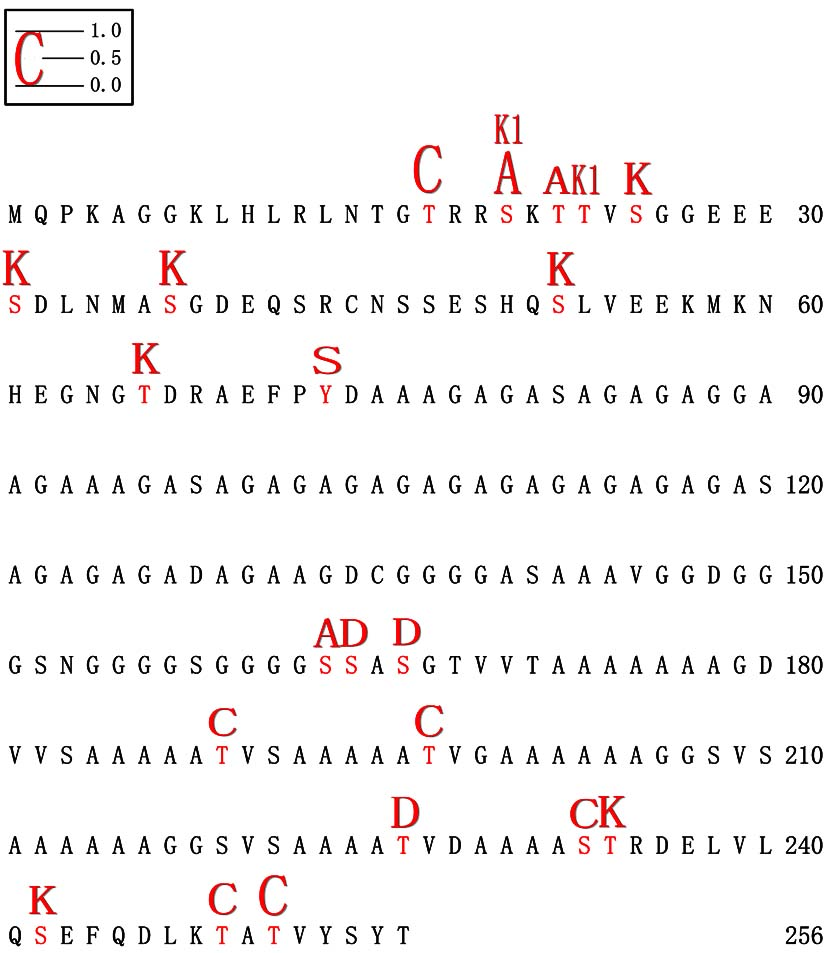
**The predicted protein kinesis and the potential position of GON-SJTU1**. The predict protein kinesis are represented by different letters with heights equal to the prediction values (0.0-1.0) and plotted along the amino acid sequence position. C: PKC; A: PKA; K: CK2; S: Src; K1: CK1; D: Cdc2.

A database BLAST search indicated that no homologues had previously been identified or studied and no conserved domain had been reported either. This rat gene was a new type, which was named *GON-SJTU1*. Several predicted phosphorylation sites mainly focused within the two terminal portions of the GON-SJTU1 protein, particularly the N terminal (Figure 3). The predicted sites for casein kinases 1, 2 (CK1, CK2), PKC and PKA were collected in N- and C-terminal portions of the proteins. Only one Src site was identified in the middle portion of the protein.

### Expression of GON-SJTU1 mRNA in all rat adult tissues tested

**Figure 4:**
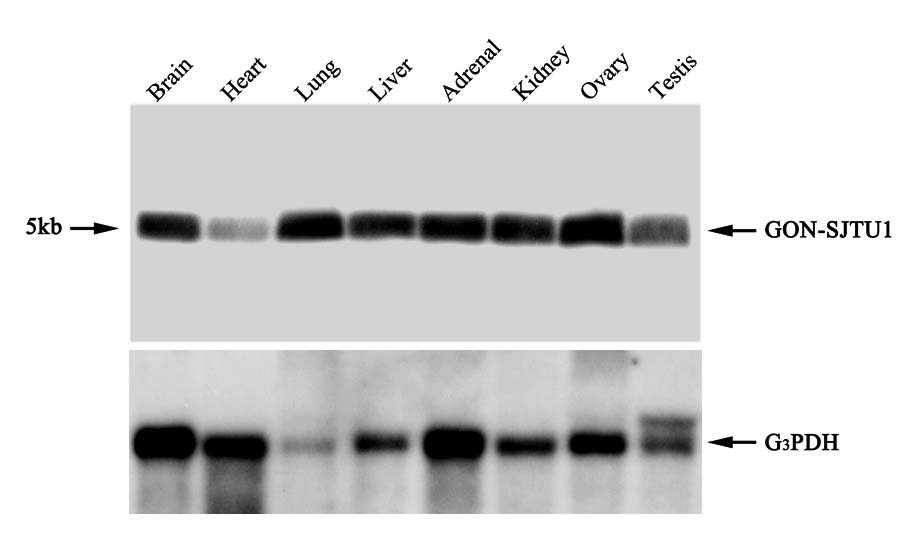
**Expression levels of *GON-SJTU1 *in multiple rat tissues**. Northern blot analysis showed that the mRNA of *GON-SJTU1 *was about 5 kb in length. The expression of this gene was detected in all tested rat tissues, brain, heart, lung, liver, adrenal, kidney, ovary and testis.

The rat *GON-SJTU1 *cDNA probe recognized a single RNA transcript of ~5 kb (Figure 4). By Northern blot analysis, *GON-SJTU1 *mRNA was detected not only in rat testes on day 60 (n = 4), but also in all of the other tissues tested (Figure 4).

### Diverse expression of GON-SJTU1 in rat testes on different days

**Figure 5:**
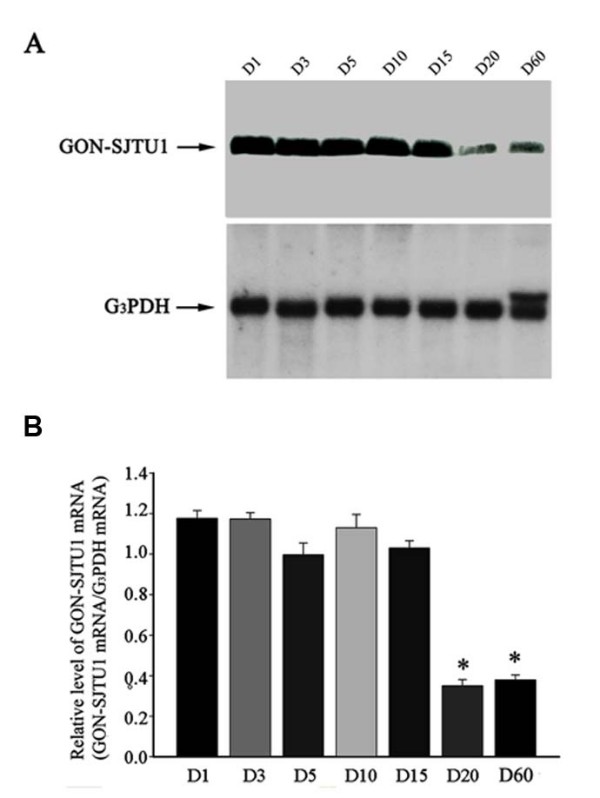
**mRNA of *GON-SJTU1 *was expressed diversely in rat testes at different ages**. (A) Representative Northern analysis of GON-SJTU1 in total RNA from samples obtained from testes on postnatal days 1, 3, 5, 10, 15, 20, or 60. Expression of G3PDH was used as an internal standard to control for evenness of loading. (B) In each of three replicate analyses, Northern blots were quantified, and the results were expressed as the ratio of GON-SJTU1:G3PDH. The bars represent the means ± SEM of the data for each age, and bars marked with star were significantly different from each other (p < 0.05).

To focus on the role of the GON-SJTU1 protein in rat testes, Northern blot analysis was performed. As shown in Figure 5, *GON-SJTU1 *expression was exhibited in an age-dependent manner in postnatal rat testis. A strong signal of its mRNA was found in the early juvenile rat (on day 1~15); however, there was a sharp decrease in its expression level when testes were obtained from 20- and 60-day-old rats (Figure 5).

**Figure 6:**
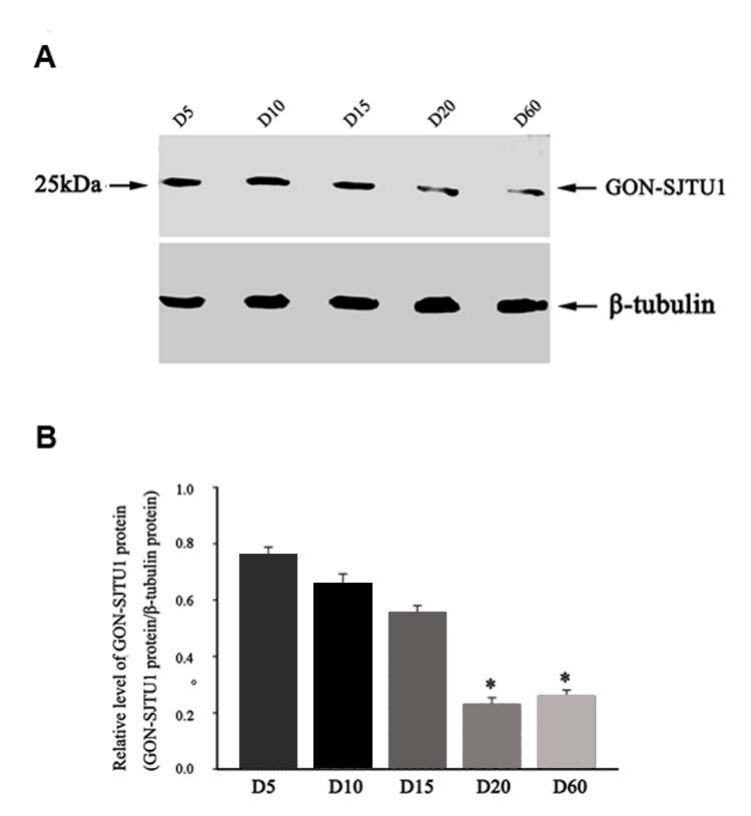
**The expression of GON-SJTU1 protein in rat testes at different ages**. (A) Representative western analyses of protein from samples obtained from testes on postnatal days 5, 10, 15, 20, or 60. The expression of β-tubulin was used as an internal standard for normalization. (B) In each of three replicate analyses, Western blots were quantified, and the results were expressed as the ratio of GON-SJTU1: β-tubulin. The bars represent the means ± SEM of the data for each age, and bars marked with star were significantly different from each other (p < 0.05).

The results of Western blotting with anti-GON-SJTU1 antibody were consistent with the results of Northern blot analysis (Figure 6).

### Expression of GON-SJTU1 in rat testis germ cells

**Figure 7:**
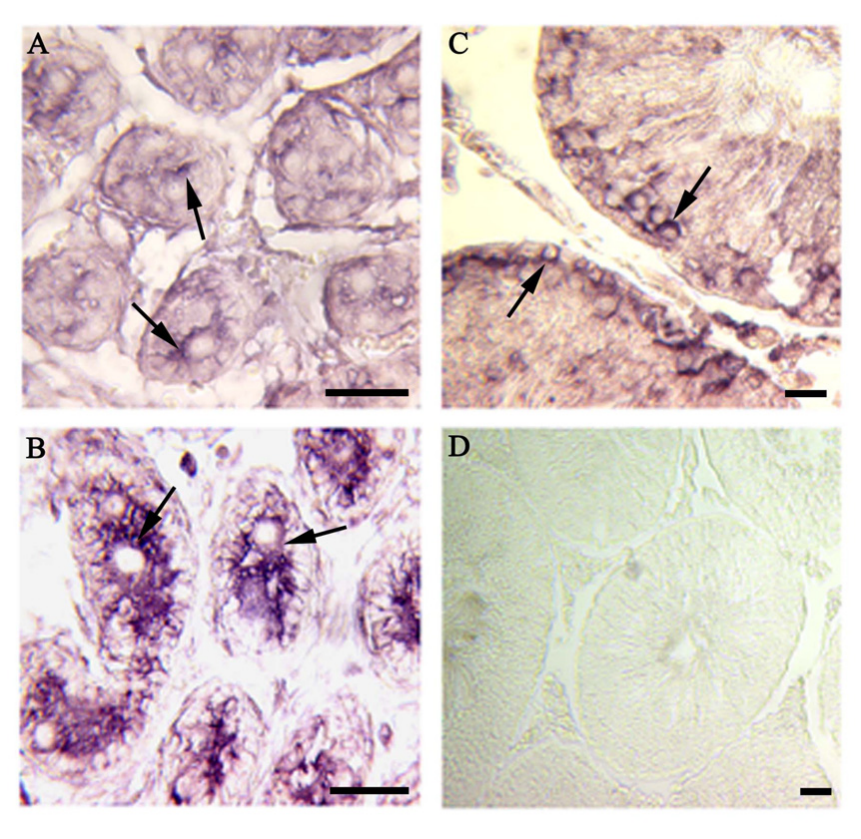
***In situ *hybridization of *GON-SJTU1 *mRNA in rat testis**. The slides of testis on day 1 (A), day 5 (B), day 60 (C) were analyzed using the DIG-labeled cRNAs probes specific for *GON-SJTU1*. The signals were observed in some germ cells (pointed with arrows). The negative control was also performed (D). Bar = 50 μm.

**Figure 8:**
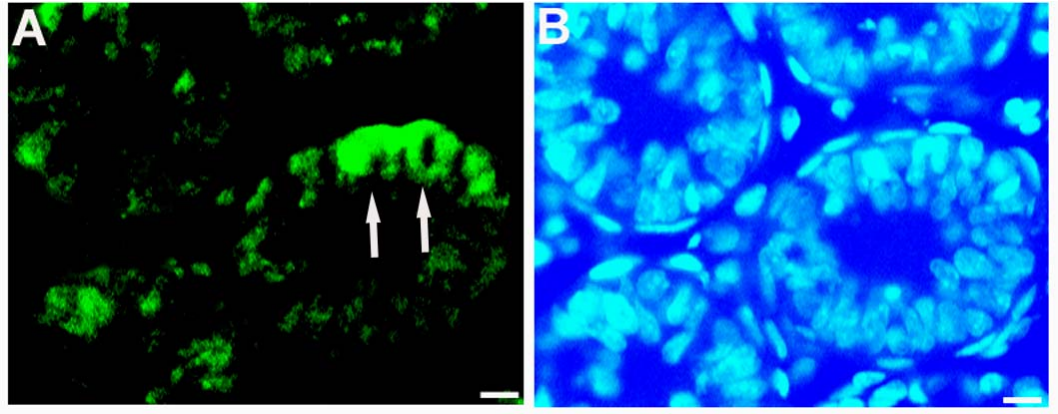
**Subcellular localization of GON-SJTU1 in rat testis on day 7 by immunohistochemistry**. GON-SJTU1 protein was detected in cytoplasm of some germ cells (green signals) (A). Corresponding DAPI image is shown in (B). Bar = 50 μm.

*In situ *hybridization with the *GON-SJTU1 *probe was performed to examine testes on days 1, 5 and 60. Within rat testis, *GON-SJTU1 *mRNA signal was present in some germ cells, especially in gonocytes and spermatogonial stem cells (Figure 7). Immunohistochemistry analysis demonstrated that GON-SJTU1 was indeed expressed in the cytoplasm of some germ cells in the 7-day testis (Figure 8).

## Discussion

A 1717 bp cDNA of *GON-SJTU1 *gene was cloned, which comprises a 771 bp ORF predictably encoding a 256-amino-acid protein. The Northern and Western blotting experiments confirmed the information mentioned above. *GON-SJTU1 *may be a new type of identified gene, as it has no homologues in the rat or any other animals. No conserved domain had been found in this protein. Only a few phosphorylation sites were predicted in its N- and C- terminal portions, most of which are predicted sites for CK2 and PKC. PKC and Src have been found to be involved in germ cell apoptosis of testis [28,29]. In addition, Src was shown to be highly expressed at stages VII-VIII [30]. CK2 is also implicated in the regulation of spermatogenesis [31,32]. The question as to whether phosphorylation or dephosphorylation of GON-SJTU1 is associated with its function, and whether PKC, CK2 or Src regulates the function of GON-SJTU1 remains to be answered in future studies.

In this study, we also analyzed the potential role of *GON-SJTU1 *in rat testis. The results showed that *GON-SJTU1 *had an age-dependent expression profile, and *in situ *hybridization analysis indicated that mRNA of *GON-SJTU1 *was expressed in some germ cells, including type A spermatogonial stem cells (Figure 7). This implied that *GON-SJTU1 *may play a role in spermatogenesis. The results of *in situ *hybridization analysis appeared to coincide with that of Northern and Western blotting using testes of 1- to 60-day-old rat: *GON-SJTU1 *products were highly expressed in one-day old rat testis, as only the germ cells present are gonocytes at this time. The high expression of GON-SJTU1 protein was constant until day 20, and showed a sharp decrease from day 15 to day 20, at which the area density and numerical density of germ cells increased to its peak [33].

Furthermore, postnatal development of rat testis has been well characterized by some researchers [5,33-38]. In one-day old animals, gonocytes are the only germ cells present; at 5 days, a mixture of gonocytes and spermatogonial stem cells are found; at day 9, type B germ cells are observed; at day 10, early spermatocytes at the preleptotene stage are apparent; at day 18-20, meiotic divisions of spermatocytes occur; at day 25, round spermatids can be observed. The gonocytes resume proliferation to give rise to type A spermatogonial stem cells shortly after birth, which marks the start of spermatogenesis. Spermatogonia are converted to primary spermatocytes but the rate increases rapidly only after day 20 [5,33,35].

Base on these data, we determined that the period of high expression of the GON-SJTU1 protein in rat testis correlated with that of early stages of rat spermatogenesis, from the transition of gonocytes to spermatogonia, to the first meiotic division of spermatocytes. This suggested that this protein played a role in these stages of spermatogenesis. However, the details of how GON-SJTU1 is involved in the rat spermatogenic process remain unknown.

The results of our study have identified the key time-point and cell types of GON-SJTU1 expression. The timing of the changes of GON-SJTU1 expression (from day 15 to day 20) is in accordance with the reduction of the rate of spermatogenic progression in rat testis, appearance of meiotic divisions of spermatocytes and the wave of germ cell apoptosis [14,15,35]. Therefore, there are at least three speculations as to how GON-SJTU1 participates in rat spermatogenesis.

First, GON-SJTU1 may be involved in regulating the rate of spermatogenic progression. van Haaster and de Rooij [35] indicated that the rate of progression of spermatogenesis was much higher before day 15 and then strongly reduced. Second, GON-SJTU1 may be involved in cell mitosis. This may also explain why GON-SJTU1 was not only expressed in the testis but also in brain, heart, liver, lung, kidney, adrenal and ovary. Third, GON-SJTU1 could relate to the survival of germ cells. The major germ cell type in apoptosis is the spermatocyte on day 20 [14]. However, the expression lever of GON-SJTU1 sharply reduced from day 15 to day 20.

GON-SJTU1 may also participate in some signal networks to regulate spermatogenesis. The expression of the *c-kit *gene has been reported in rat gonocyte from the day of birth to day 5. This protein has been indicated to be essential for the migration of gonocytes, which is crucial for their survival [39,40]. In rats, both mRNA and protein levels of c-kit are highly detected in differentiating spermatogonia, types A1 to A4, and are persistent in low levels in meiotic pachytene spermatocytes [41-43]. Some Bcl-2 family members also present an age-development expression pattern. Both Bax and Bcl-w expression levels peak on day 20 and decrease thereafter [14]. It remains to be tested whether GON-SJTU1 takes part in the SCF/c-kit signaling system and regulates the function of Bax or Bcl-w. Our studies show that GON-SJTU1 might play a role in spermatogenesis, but the exact details of this role require further study, such as GON-SJTU1 knockout mice are generated and analyzed.

## Conclusion

*GON-SJTU1 *can express in some male germ cells and may play a role in the biological process of spermatogenesis. The exact mechanism with GON-SJTU1 in the spermatogenesis needs to be clarified in further study.

## Competing interests

The authors declare that they have no competing interests.

## Authors' contributions

ZY, NS, SW, and GGT carried out the experiments. ZY drafted the paper. JW initiated and mentored the study in addition to providing a valuable framework for drafting the paper. All authors read and approved the final manuscript.
